# Visual and psychological stress during computer work in healthy, young females—physiological responses

**DOI:** 10.1007/s00420-018-1324-5

**Published:** 2018-05-30

**Authors:** Randi Mork, Helle K. Falkenberg, Knut Inge Fostervold, Hanne Mari S. Thorud

**Affiliations:** 1 Department of Public Health Science, Norwegian University of Life Sciences, Ås, Norway; 2 Department of Optometry, Radiography and Lighting Design, University of South-Eastern Norway, National Centre for Optics, Vision and Eye Care, P.O. Box 235, 3603 Kongsberg, Norway; 3 Department of Psychology, University of Oslo, Oslo, Norway

**Keywords:** Glare, Psychological stress, Trapezius, Computer work, Vision, Posture

## Abstract

**Purpose:**

Among computer workers, visual complaints, and neck pain are highly prevalent. This study explores how occupational simulated stressors during computer work, like glare and psychosocial stress, affect physiological responses in young females with normal vision.

**Methods:**

The study was a within-subject laboratory experiment with a counterbalanced, repeated design. Forty-three females performed four 10-min computer-work sessions with different stress exposures: (1) minimal stress; (2) visual stress (direct glare); (3) psychological stress; and (4) combined visual and psychological stress. Muscle activity and muscle blood flow in trapezius, muscle blood flow in orbicularis oculi, heart rate, blood pressure, blink rate and postural angles were continuously recorded. Immediately after each computer-work session, fixation disparity was measured and a questionnaire regarding perceived workstation lighting and stress was completed.

**Results:**

Exposure to direct glare resulted in increased trapezius muscle blood flow, increased blink rate, and forward bending of the head. Psychological stress induced a transient increase in trapezius muscle activity and a more forward-bent posture. Bending forward towards the computer screen was correlated with higher productivity (reading speed), indicating a concentration or stress response. Forward bent posture was also associated with changes in fixation disparity. Furthermore, during computer work per se, trapezius muscle activity and blood flow, orbicularis oculi muscle blood flow, and heart rate were increased compared to rest.

**Conclusions:**

Exposure to glare and psychological stress during computer work were shown to influence the trapezius muscle, posture, and blink rate in young, healthy females with normal binocular vision, but in different ways. Accordingly, both visual and psychological factors must be taken into account when optimizing computer workstations to reduce physiological responses that may cause excessive eyestrain and musculoskeletal load.

## Introduction

Computers and other electronic devices are now widely used during both occupational and leisure activities. Among computer workers, there is a high prevalence of visual complaints related to the visual stress associated with intensive near-visual work (Ranasinghe et al. 2016; Rosenfield 2011; Woods 2005). Pain in the neck and shoulder area is also prevalent among individuals who work with computers (Gerr et al. 2002; Mohanty et al. 2017; Woods 2005), but the mechanisms inducing neck and shoulder pain during computer work are not fully understood (Andersen et al. 2011; Jun et al. 2017; Ortego et al. 2016; Wærsted et al. 2010). Research has shown that during computer work, visual discomfort and pain in the neck and shoulder area are associated, indicating a relation between the visual and the musculoskeletal system (Fostervold et al. 2006; Helland et al. 2008; Richter et al. 2011; Wiholm et al. 2007). This yet highlights the importance of visual ergonomics and optimization of visual conditions to prevent both visual and musculoskeletal discomfort among computer workers.

Based on an evolutionary stress model, Fostervold et al. (2014), described the link between vision, oculomotor factors, and the musculoskeletal system as adaptive and functional. In this model, a key element is the notion of evolutionarily-novel environments: environments departing from those for which the human species has developed specific phenotypic adaptations. Although such adaptations may seem functional, ongoing efforts to cope in evolutionary-novel environments may give rise to new problems. Subjective complaints and ailments associated with computer work are in this context seen as a mismatch between species-specific adaptations to vision at close distance and demands imposed by the computer work environment (Fostervold 2003; Fostervold et al. 2006; Lie et al. 2000).

Near-visual tasks, such as computer work, are static and often of long duration. This places a high demand on both smooth and cross-striated muscles in and around the eyes (Lie and Watten 1994; Rosenfield 2011). The eyes focus on near objects by accommodating (contracting the ciliary muscle to make the lens more spherical), by converging (two of the extraocular muscles, m. rectus medialis, rotate the eyes inward to keep single vision), and by miosis (contracting the iris sphincter muscle to reduce pupil size to increase visual acuity) (Levin et al. 2011). Activation of muscles in the neck and shoulder area during near-visual work has also been shown, and it is likely due to the gaze stabilization necessary for maintaining a clear picture on the retina (Bizzi et al. 1971; Lie et al. 2000; Lie and Watten 1987; Richter and Forsman 2011). Increased visual stress during near-visual work, such as inadequate lighting (e.g., glare), uncorrected refractive errors, and accommodative and binocular disorders, puts extra stress on the visual system and head-stabilizing musculature, and may aggravate symptoms from the eyes and the neck and shoulder area (Gowrisankaran and Sheedy 2015; Rosenfield 2011). In addition, glare has been shown to affect subjects with normal binocular vision during computer work, resulting in decreased reading speed/productivity (Glimne et al. 2015), increased fixation disparity variation (Glimne et al. 2013), and increased muscle blood flow in the trapezius (Mork et al. 2016).

Glare has always been a potential risk factor in the human environment and should therefore not be considered evolutionarily novel. In this regard, glare is comparable to other environmental stressors, where a general stress response would be expected. The effect of glare varies from hardly noticeable to detrimental for functional vision and the visual system contains several adaptations aimed at reducing the negative impact of glare. Examples could be: the anatomical design of the face with the eyes placed within the orbital cavity to shield for overhead light, increased eyelid squinting (Mork et al. 2016; Sheedy et al. 2003), increased blink rate (Gowrisankaran et al. 2007; Nahar et al. 2007), and pupillary contraction (Ellis 1981). Behavioral countermeasures like changing posture, looking away, or shielding the eyes from the bright light source is also often used during glare conditions (Boyce 2014). These adaptations and countermeasures often eliminates or reduce the effect of glare in ordinary environmental settings. In contrast, modern computer work environments represents a problem with a variety of artificial light sources, surfaces with high specular reflection, and static intensive near-work with high gaze angle and reduced possibilities to change position to avoid the glare sources. Thus, glare during computer work may accordingly be considered as evolutionarily novel as traditional adaptations and countermeasures may not suffice in such environments. According to the evolutionary stress model, continuing computer work with glare exposure may thus initiate increased load on the worker leading to negative consequences and discomfort.

Increased psychological load has been shown to induce different physiological responses in humans; such as increased activity in the sympathetic nervous system, increased heart rate, decreased blink rate, and increased muscle activity and blood flow in trapezius and facial muscles (Hidaka et al. 2004b; Larsson et al. 1995; Lundberg et al. 2002; Nilsen et al. 2007; Rodriguez et al. 2018; Skoluda et al. 2015). Some similar responses have been found to be induced by excessive light/glare, a visual stressor (Belkić 1986; Mork et al. 2016; Saito et al. 1996), suggesting that effects seen during exposure to visual stress also may involve a central mediated stress response.

However, few studies have investigated both adaptations to glare and reactions to stress in computer work environments. The aim of the present study was thus to elucidate this relationship by means of the two common occupational stressors: visual stress (direct glare) and psychological stress. Based on previous research and deliberations from the evolutionary stress model the following hypotheses were tested: (1) the trapezius and orbicularis oculi muscles are affected by glare and psychological stress. (2) Glare and psychological stress affect cardiovascular responses. (3) Glare and psychological stress induce adjustments in sitting posture and visual parameters.

## Methods

### Design and procedures

The study was a laboratory experiment applying a counterbalanced, fully factorial, repeated 2 × 2 × 4 design. The within-subject conditions were visual stress (direct glare) (on/off), psychological stress (on/off), and four time points [rest, 5, 10 min and recovery (see “Data analysis”)]. To test the impact of the stress conditions, four 10-min computer-work conditions were performed in random order, each of which contained the same task but with different stress requirements, as follows.

#### Low stress (LS)

In the LS condition, the participants worked with appropriate lighting (glare source turned off) and minimal psychological stress. The participants were encouraged to perform the computer task as well as possible, but it was emphasized that their performance would not have a major influence on the test outcome.

#### Visual stress (VS)

In the VS condition, the participants worked with exposure to excessive lighting (glare source turned on) and minimal psychological stress as in LS. See separate section: glare source and lighting.

#### Psychological stress (PS)

In the PS condition, the participants worked with appropriate lighting (glare source turned off), and exposure to psychological stress. A combination of three stress-inducing procedures was used: (1) the participants were told to work as fast and accurately as possible and that their performance would have a major influence on the test outcome (time and precision pressure). (2) The participants were told that, after the condition, they would have to answer questions from the text they had read (social-evaluative threat). (3) A visible video camera was turned on to monitor the participants throughout the computer-work session (social-evaluative threat).

#### Visual and psychological stress (VPS)

In the VPS condition, the participants worked with both exposure to excessive lighting (glare source turned on) and psychological stress as described in VS and PS.

For each participant, the order of the four computer-work conditions were drawn at random from predetermined envelopes to control for possible order effects (counterbalanced design). In all four conditions, the participants accomplished the same computer task: they read a text on a computer screen, identified spelling errors in the text, and marked these errors in bold using a regular wireless laser mouse as a pointing device. The text was in Norwegian and there was on average one spelling error on every third line (unevenly distributed). The examiner was the same for all participants.

**Fig. 1:**
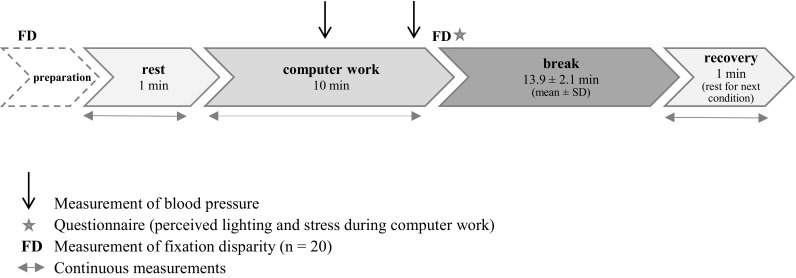
Flowchart of the first test condition. Muscle activity, muscle blood flow, heart rate, blink rate (in PS and VPS) and postural angles were recorded continuously during rest, computer work, and recovery. Blood pressure was registered twice during the computer task (at 4 and 9 min). Fixation disparity was measured during the preparation period (baseline) and immediately after the 10-min computer-work session. Directly after the fixation disparity measurement, the participants completed the questionnaire regarding perceived lighting and stress. All four conditions included the same parts as shown in the figure (except preparation), and PPG rest recording before the first condition was used as the baseline for PPG results in all conditions

All four computer-work conditions consisted of (1) a 1-min rest recording before computer work (*rest*), (2) 10 min of computer work (LS, VS, PS or VPS), (3) a break [13.9 ± 2.1 min (mean ± SD, *n* = 43)], and (4) a 1-min rest recording after the break to measure the recovery (*recovery*). The recovery period at the end of one condition constituted the rest period of the following condition (Fig. 1).

During all rest recordings (rest and recovery), the participants were instructed to sit in a comfortable position with their hands in the lap, relaxing the shoulder and neck muscles, and with a comfortable gaze towards an eye-height distance target (approximately 6 m away), relaxing their eyes. To avoid use of the orbicularis oculi muscle, participants were not allowed to close their eyes during the rest recordings.

During breaks between conditions (no measurements), all participants were instructed to rest in a sitting or standing position. Because of the attached equipment, participants could stand up, but their walking range was limited. To rest their eyes, they could choose to close their eyes or look around freely, but they were not allowed to perform any visually demanding activities, such as looking at their cell phones. To make breaks as similar as possible for all participants, a radio played pop music and talk with the examiner was minimized. The glare source was turned off during all rest recordings and during breaks.

All participants got verbal and written information about the study before giving their informed consent. However, all were naiive to the specific aim or expected outcomes of the study to avoid respondent bias.

All information given about the study was standardized and written down. The participants did not know the condition order beforehand and were informed of it verbally just before the beginning of each computer-work session. In a pre-meeting or when attending the lab, all participants performed a short computer-work session to familiarize themselves with the proofreading task. After finishing the testing procedure, all participants were debriefed and, to ensure that new participants were naive observers, instructed not to tell other participants details about the experiment.

All participants attended the lab once, between 8:00 a.m. and 12:00 p.m., and the visit lasted for approximately 3 h. The first hour was used for information, completing questionnaires, initial measurement of fixation disparity (baseline), and connection and calibration of the measurement equipment. The testing procedure lasted for about 2 h.

### Participants

The sample consisted of 43 healthy female undergraduate students recruited from the University College of Southeast Norway (Table 1). Sample size was calculated a priori with a test power of 80%, a significance level of 5% (two-tailed) and standard errors based on trapezius photopletysmography measurements from Mork et al. (2016) (Owen 1962). Trapezius muscle blood flow was a main variable to investigate in the current study, and the power analysis showed that 36 participants should suffice to identify a 15% difference in trapezius blood flow between two repeated means, without and with glare exposure.

**Table 1:** Descriptive characteristics of the participants

	Mean ± SD	*N*
Subjects		
Age	21.4 ± 2.4 years	43
Experience with computers	10.3 ± 2.5 years	43
Use of electronic devises (per day)	5.2 ± 2.5 h	43
Visual status		
Distance visual acuity, LogMAR	− 0.2 ± 0.1	43
Spherical equivalent, right eye	− 0.8 ± 1.6	43
Binocular accommodation amplitude, R.A.F-rule^a^	11 ± 2D	42
Near point of convergence, R.A.F-rule^a^	5 ± 1 cm	43
Horizontal phorias at distance, Cover test	− 0.6 ± 1.4Δ	43
Horizontal phorias at near, Cover test	− 1.8 ± 2.3Δ	42
Stereo acuity, TNO	50 ± 16ʺ	43
Fixation disparity, the Sheedy Fixation Disparometer (60 cm on test day)	− 1.6 ± 4.4 arcmin	20
Personality		
Positive trait personality, PANAS (sum score)	16 ± 2	42
Negative trait personality, PANAS (sum score)	8 ± 3	43

^a^Measurement was performed by the R.A.F. near-point rule (Neely 1956)

Data were collected in two different winter periods (December–February): 24 women were tested in 2015 and 20 in 2016. One participant was excluded from the first period because of the exclusion criteria. Only women were included in the study, because females have shown a higher prevalence of both musculoskeletal pain (Larsson et al. 2007; Paksaichol et al. 2012) and visual symptoms during computer work (Ranasinghe et al. 2016) compared to males. In addition, females and males respond differently to stress (Collins and Frankenhaeuser 1978; Luine et al. 2017).

The participants underwent an optometric examination at National Centre for Optics, Vision and Eye care, Kongsberg, Norway, prior to testing to ensure normal, or corrected to normal, vision (Table 1). Twenty participants used no vision correction during testing, 16 wore glasses, and seven used contact lenses. All participants had normal vision functions and good eye health. None of the subjects had vertical phorias or uncompensated fixation disparities. Exclusion criteria were chronic pain in the neck and shoulder area the last 6 months, history of eye trauma and surgery, dyslexia, mental illness, and systemic disease/regular use of medications affecting circulation, pain sensation, vision, or visual comfort.

The participants completed two questionnaires on the testing day (Table 1). The first questionnaire recorded information about visually demanding work prior to attending, coffee or alcohol drinking or exercise during the previous 12 h, hours slept the previous night, medications taken the same day, and smoke/snuff use (i.e., smokeless tobacco made from ground or pulverized tobacco leaves). The second questionnaire recorded information about personality traits by means of the 10-item mood scale, Positive and Negative Affect Schedule PANAS (Watson et al. 1988). This form rated how the participants usually felt according to five negative and five positive personality traits, scoring each specific trait from 1 (nothing) to 5 (much). Dispositional tendencies to experience negative emotions have repeatedly been identified as an important factor in the process of appraising environmental conditions at work (Girardi et al. 2011). Individuals dominated by negative affect have shown higher autonomic activation compared to individuals dominated by positive affect (Kreibig 2010). To control for this confounder, negative and positive affect (sum score of five negative/positive items) were included as covariates in the statistical analyses.

Seven participants performed short periods of visually demanding work (e.g., reading, computer work) for more than 2 h before the start of testing. Eight of the participants had drunk 1–2 cups of coffee 3–4 h prior to testing, two had drunk 1–2 units of alcohol the evening before attending, nine were habitual smokers or snuff users, and eight had smoked or used snuff earlier on the testing day. None of the participants had exercised during the previous 12 h, and they had slept 7.2 ± 1.8 h (mean ± SD, *n* = 43) before testing.

### Workstation

The participants were seated in a stationary office chair without wheels. Distance to the screen, gaze angle, underarm support, screen tilting, and height of the desktop and chair were individually optimized according to national and international recommendations (Arbeidsplassforskriften 2011, ISO 9241-5 1998; Lillelien et al. 2012). Distance to the screen was 65 ± 6 cm (mean ± SD, *n* = 42). The gaze angle, measured as the angle between the midpoint of the readable window on the screen and an imaginary horizontal line at eye level, was 21 ± 2° (mean ± SD, *n* = 42) downward. Downward gaze angle has been shown to be beneficial during computer work (Fostervold et al. 2006). The participants were allowed to move freely within normal ranges during the conditions. The sitting position was controlled for by measuring postural angles. Before the experiment, the participants were informed that they could be told to return to the initial optimal position if they moved into very unfavorable ergonomically postures (e.g., leaning extremely forward or resting the head in one of their hands). This was done to minimize the possibility of ergonomically loads and significantly alternations in viewing distance to the computer screen. To minimize mental/visual disturbances during the conditions, the examiner sat 3 m away and outside of the participants’ visual field.

All computer work was performed on an external 24ʺ anti-reflection LCD-screen with 1920 × 1200 pixels resolution and a mean refresh rate of 69.5 Hz (HP LA2405x), connected to a closed laptop. The brightness of the external monitor was 60% of maximum. The font size was 12 point Times New Roman, with a letter size of 3 mm (Capital E). This corresponds to a vertical viewing angle of 0.26° at a viewing distance of 65 cm. To minimize individual differences in light exposure and neck angle due to viewing angle, the readable window was reduced to the upper half of the screen. The background behind the readable window was light gray. The ambient air temperature and relative humidity was 22 ± 1 °C and 38 ± 9% (mean ± SD, *n* = 42).

### Glare source and lighting

The glare source consisted of two large luminaries centered 60 cm behind the computer screen. When turned on, the luminaires simulated an office window exposing the participants to excessive light (visual stress) during computer work. The luminaires consisted of translucent acrylic diffusing fronts (1.25 m × 0.57 m), equipped with six fluorescent tubes (T5, F28W/830), and the luminous intensity of the luminaries was 4634 ± 749 cd/m^2^ (mean ± SD, measured across the screens), range 3230–5870 cd/m^2^.

The luminance levels with the glare source turned off were 155 cd/m^2^ in the working field (computer screen turned on), 90 cd/m^2^ in the immediately surrounding area (desktop), and 61 cd/m^2^ in the background area. This is within the recommended luminance ratio 5:3:1 for a workstation (Anshel 2007). With the glare source turned on, the luminance levels were 155, 520, and 4634 cd/m^2^, respectively, and the luminance ratio was therefore 1:3:30. The illuminance on the desktop between the participant and the computer screen was approximately 1500 and 659 lx with the glare source turned on and off, respectively. A Hagner Universal Photometer (Modell S4, Sweden) was used for the luminance measurements, whereas a Hagner Digital Luxmeter (Model EC1, Sweden) was used to measure illuminance.

### Measurements

Muscle activity in dominant m. trapezius, muscle blood flow in dominant m. trapezius and in m. orbicularis oculi (dominant eye), heart rate, blink rate, and postural angles were registered continuously during computer-work and rest recordings (Fig. 1). Blood pressure was registered twice during each condition (after 4 and 9 min). Fixation disparity was measured immediately after each 10-min computer-work period, and directly after that, the participants completed the questionnaire regarding workstation lighting and stress during computer work.

The orbicularis oculi muscle is a thin elliptical muscle surrounding the eye, extending from the lids to the brow, temple, and cheek. This muscle consists of two main parts: the palpebral (inner) part, responsible for involuntary and voluntary blinking, and the orbital part, which closes the lids firmly (Bron et al. 1997; Thorud et al. 2012). During eye squinting, the orbital part contracts as the palpebral part relaxes.

The trapezius muscle is a large, superficial muscle that extends from the occipital bone, the cervical and the thoracic region, and laterally to the spine of the scapula. Trapezius consists of three functional parts: an upper (descending) part, a middle region (transverse), and a lower (ascending) part. The actions of the upper descending part are elevating the scapula (supporting the weight of the arm) and assisting with the stabilization and movement of the cervical spine (Johnson et al. 1994).

#### Muscle activity in m. trapezius

Muscle activity was measured unilaterally in the dominant trapezius (upper, descending part) during computer work using surface electrode electromyography (EMG). The muscle activity (and postural angles) measurements were carried out using a field-portable apparatus (Physiometer PHY-400, Premed A/S, Oslo, Norway) connected to a computer. The EMG signal was normalized by calibrating the EMG response to force [given as % maximum voluntary contraction (MVC)], using a calibration platform with a force transducer (Aaras and Ro 1997). Further description of the EMG method and establishing of the EMGrms/force relationship for the actual range of work load below 30% MVC can be found in Aaras et al. (1996) and Mork et al. (2016).

#### Muscle blood flow in m. trapezius and m. orbicularis oculi

Muscle blood flow was measured unilaterally on the orbital part of the orbicularis oculi (dominant eye) and on the upper descending part of the trapezius (dominant side) using photoplethysmography (PPG). PPG is a noninvasive optical technique for continuous monitoring of muscle blood flow and can be used to detect blood volume changes in the microvascular bed of muscle tissue (Sandberg et al. 2005; Zhang et al. 2001). In this study, two custom-designed optical probes (Department of Biomedical Engineering, Linköping University, Sweden) were used. The probes were attached on the orbital part of the orbicularis oculi below the participant’s dominant eye and medial to the EMG electrodes on the upper descending belly of the trapezius. The PPG method is described in Thorud et al. (2012), and Mork et al. (2016).

#### Postural angles

Postural angles were measured continuously during both the rest and computer-work periods using two dual axis inclinometers connected to a physiometer (Premed A/S, Oslo, Norway) (Aaras and Stranden 1988). The inclinometers were attached to the upper back and to the back of the head. The inclinometers were calibrated (zero value) as the participants sat in a reference body position, a balanced position without a backrest and with the arms hanging down by the sides, looking at a point at eye height at a distance of approximately 6 m. Back and head angles were measured in terms of deviation from this reference position. Flexion (leaning forward) was given as positive values, extension (leaning backward) as negative values. For the angles of lateral flexion (side bending), negative values reflected movements to the left of the reference position, and positive values reflected movements to the right. If the participants changed their viewing distance during reading, this was reflected in the measured back angles.

#### Heart rate

Heart rate during each rest session and during computer work was measured continuously by the PPG method. The AC component of the PPG signal is synchronous with the heart rate (Lindberg and Oberg 1991), and heart rate was estimated by counting the number of heartbeats (AC-peaks) per minute.

#### Blood pressure

Systolic and diastolic blood pressure were assessed from the nondominant upper arm after 4 and 9 min of computer work using an automatic oscillometric blood flow monitor (A & D Medical, Model UA-767Plus 30). The artery position mark was placed 1–2 cm above the elbow, in a medial position on the upper arm (in line with the ring finger with a supinated forearm). Before the start of testing, the participants were asked not to think of the blood pressure measurements, but rather to stay focused on the computer task. To familiarize participants with the equipment, one measurement was performed during the preparation phase.

#### Blink rate

One of the stress-inducing procedures in the conditions with psychological stress exposure (PS and VPS) was a visible video camera to monitor the participants during computer work. The videos were used to manually count blink rate (blinks/min). The overall analysis of blink rate during computer work included data for mean blinks in the first half (0–4 min) and the second half (5–9 min) of the conditions, and ‘computer work’ reported in Fig. 7a is therefore the average of these measuring points. In addition, blink rate in the first minute of the computer-work period (0–1 min, ‘start of the work session’) was analyzed with respect to ‘the rest of the work session’ (average of blinks/min in 2–9 min, with the 4th min excluded) (Fig. 7b). The video camera was not used in the LS and VS conditions to avoid adding psychological stress. Hence, blink rate data were only available from PS and VPS.

#### Fixation disparity

Fixation disparity are small misalignments of the visual axis during binocular vision, without causing double vision. The misalignments may be vertical, horizontal, or both, and are normally compensated by the visual system. In the case of horizontal fixation disparity, as measured in the current study, the fixation disparity is a vergence error defined as either an exo-disparity or eso-disparity, where the visual axis fixates behind or crosses in front of the fixation point. Small fixation disparities (minutes of arc) are common in subjects with normal binocular vision (Jaschinski 2017). Yet, alternations in fixation disparity is considered an indicator of oculomotor stress (Glimne et al. 2013; Pickwell et al. 1987).

Horizontal fixation disparity was only measured in the second test period (*n* = 20). Immediately after completing the 10-min computer-work period, the participants turned their chair around, put on a pair of polarized glasses, and placed their head in a chin-and-head support facing a Sheedy fixation disparometer (Dwyer 1982). The test distance was approximately the same as during the computer-work periods, 60 cm. A Sheedy fixation disparometer consists of successive pairs of vernier lines of increasing angular separation within a structureless field, each line being viewed by one eye through the polarizing glasses. The participants were asked to read the text on the disparometer in order focus the eyes on the correct distance before measuring the fixation disparity. The disparometer was adjusted to zero disparity at start, and was then adjusted until the participants reported that the lines appeared to be aligned. One fixation disparity measurement before the start of testing, called the baseline, was also accomplished (Fig. 1). Variation in disparity after each condition relative to baseline is called FD_change_. This value reflects the change in fixation disparity for each participant from their own baseline (0), a change in either an eso-direction (+) or exo-direction (−). Therefore, the statistical analyzes of the fixation disparity measurements were conducted with both the measured fixation disparity value and FD_change_ for each condition, both measured in minutes of arc.

#### Perceived stress and experience of the lighting

To measure how participants perceived the induced stressors in the different conditions, they were asked to report how they experienced the lighting and how stressed they felt during the computer-work periods. The questions were posed immediately after the fixation disparity measurement in each condition, and recorded on 100 mm Visual Analogue Scales (VAS) (Price et al. 1994). The left endpoint (0 mm) represented no stress/very comfortable lighting, whereas the right endpoint (100 mm) represented highly stressed/very uncomfortable lighting.

#### Work performance

Productivity was defined as reading speed during 10 min of computer work in each condition (number of words read in 10 min). Accuracy was defined as the percentage (%) of correctly marked spelling errors (correctly marked errors divided by the actual number of errors in the text read).

### Data analysis

The sampled signals of muscle activity and postural angles were ranked to produce an amplitude distribution function, ADF (Jonsson 1982). Static and median load were defined as ADF levels 0.1 and 0.5, respectively. Muscle blood flow and heart rate was recorded at a sampling frequency of 240 Hz, and was analyzed using software developed at Department of Biomedical Engineering, Linköping University, Sweden (Aqua) and MatLab R2009b (The MathWorks, Inc., US). The data analysis of muscle blood flow and heart rate is described in Thorud et al. (2012).

For all rest and recovery recordings of the continuous measurements, data were analyzed as an average value of the 1-min registrations. To exclude the possibility for the blood pressure measurements registered after 4 and 9 min during the computer-work periods to affect the results, data for the continuous measurements captured in the periods 4–5 and 9–10 min were left out of the analysis. In the analysis, either two time points, average of 0–4 min (called 5 min) and average of 5–9 min (called 10 min), or one average value for the entire work period (called *computer work*) was used. Some of the tables and graphs included only *computer work* to make the description of the results easier to follow.

Because of technical problems with the photoplethymography measurements, especially during the first test period, we obtained complete data only for 32 and 23 participants for trapezius and orbicularis oculi muscle blood flow, respectively.

### Statistics

Statistical analyses were performed in IBM SPSS Statistics (Version 24, US). The overall statistical analysis was performed with ANOVA Repeated Measures (factorial repeated 2 × 2 × 4 design). Planned contrasts were used to compare conditions if the overall analysis indicated either main effects or interaction effects. Inspection of the variables revealed that several variables departed from the normal distribution, and base-10 logarithm transformation was executed on these variables. For variables with normal distribution, untransformed data were used in the analysis. For most ANOVA analyses, Mauchly’s test indicated a violation of the assumption of sphericity. The Greenhouse Geisser correction was therefore used if nothing else is reported in the results. Untransformed data were presented in the figures, except for the blood flow data, which was shown as percentage muscle blood flow increase relative to baseline. Correlation analysis (Pearson) was performed on transformed data. An overall analysis of variance (ANOVA) was performed to investigate potential overall time effects (test order effects) throughout the experiment independent of condition.

## Results

### Analysis of covariates

Personality measures of negative and positive affect were entered as covariates in the analysis. The results did not show any significant interaction effects. Thus, personality seems not to have affected the measured variables in the current study. The co-variates were consequently discarded from further analyses.

### Experience of visual and psychological stress

**Fig. 2:**
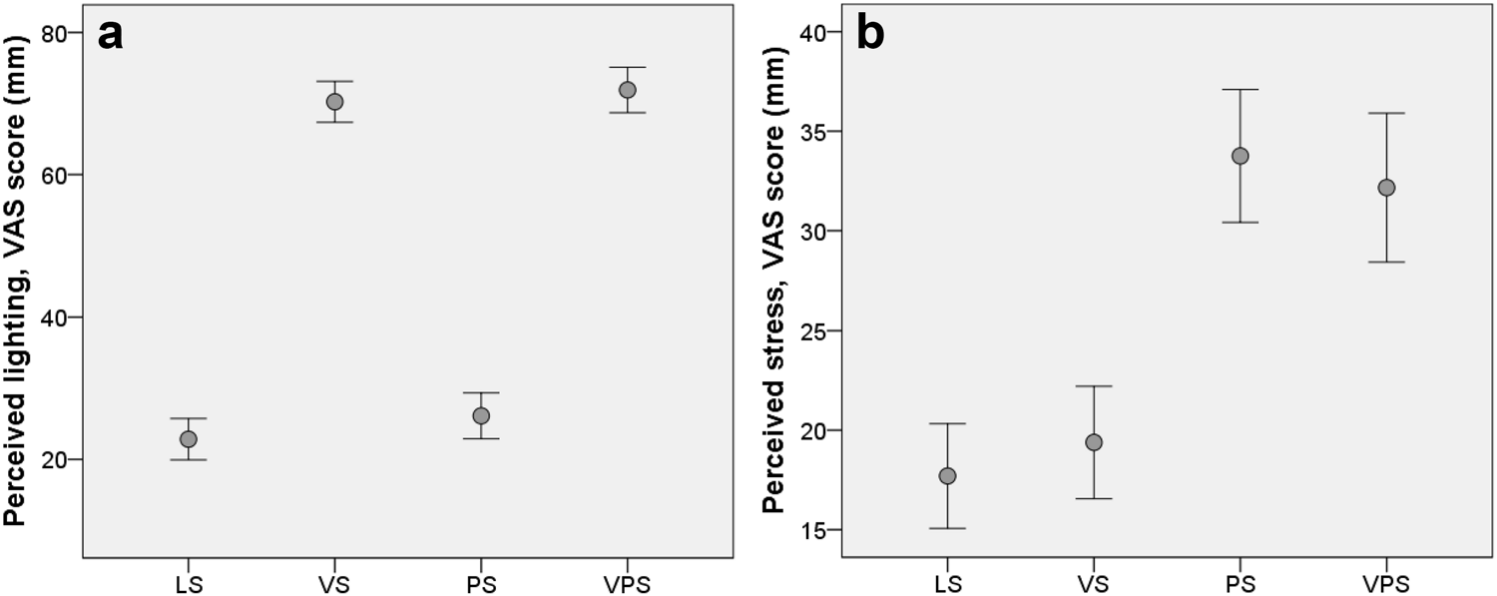
Scores (mm VAS) for **a** how participants perceived the workstation lighting, and **b** the degree of stress perceived by participants during the four computer-work conditions. *LS* Low stress, *VS* visual stress, *PS* psychological stress, *VPS* psychological and visual stress. Higher scores indicate **a** a higher degree of perceived unpleasantness of the lighting, and **b** a higher degree of perceived stress. Results are given as mean ± SEM, *n* = 43

The participants were exposed to visual stress (direct glare) in two conditions (VS and VPS) and psychological stress in two conditions (PS and VPS). The results showed a significant main effect of glare on perceived lighting during computer work; *F*(1, 42) = 84.51, *p* < .001, *η*^2^ = 0.67. Figure 2a shows that when the participants were exposed to direct glare during the computer-work period, they experienced the lighting at the workstation as significantly more unpleasant than when they were given appropriate lighting.

The analysis also showed a significant main effect of exposure to psychological stress on perceived stress; *F*(1.0, 42.0) = 29.90, *p* < .001, *η*^2^ = 0.42. This indicates that the participants felt more stressed during psychological stress exposure compared to the conditions with no psychological stress (Fig. 2b).

Furthermore, there was a significant and positive correlation between perceived stress and lighting in VPS (*r* = .392, *p* = .009), indicating that perceiving more unpleasant ambient lighting was associated with feeling more stressed. This association was not present during the other conditions.

### Muscle blood flow

#### Trapezius

**Fig. 3:**
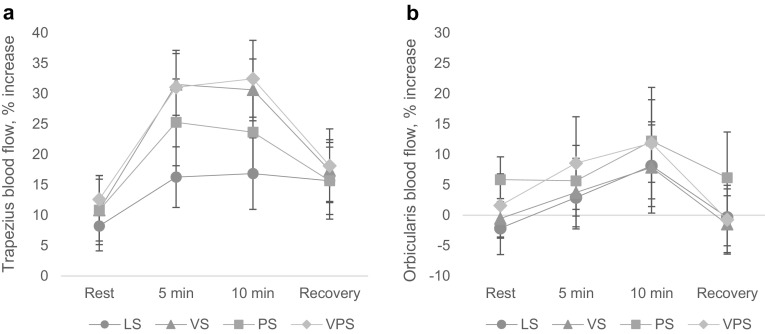
Muscle blood flow in **a** trapezius (*n* = 32) and **b** orbicularis oculi (*n* = 23) during the four computer-work conditions. *LS* Low stress, *VS* visual stress, *PS* psychological stress, *VPS* psychological and visual stress. Results are given as percentage increase in muscle blood flow relative to the initial resting value (baseline). *Rest* = rest recording before computer work; *Recovery* = rest recording after a 14-min break (see Fig. 1). All results are given as mean ± SEM

Visual stress induced increased trapezius muscle blood flow during computer work (Fig. 3a). The results showed a significant glare by time interaction on trapezius blood flow; *F*(2.50, 77.60) = 3.93, *p* = .016, *η*^2^ = 0.11. During glare exposure, blood flow at 5 min; *F*(1, 31) = 4.98, *p* = .033, *η*^2^ = 0.14, and 10 min; *F*(1, 31) = 4.39, *p* = .045, *η*^2^ = 0.12, was significantly higher than during the rest period. Blood flow during recovery was not significantly different from that during rest.

In addition, the results showed a significant main effect of time on trapezius blood flow; *F*(1.89, 58.68) = 18.84, *p* < .001, *η*^2^ = 0.38. Overall, blood flow at 5 min; *F*(1, 31) = 35.94, *p* < .001, *η*^2^ = 0.54, at 10 min; *F*(1, 31) = 27.45, *p* < .001, *η*^2^ = 0.47, and during recovery; *F*(1, 31) = 6.95, *p* = .013, *η*^2^ = 0.18, was higher compared to that during rest in all conditions.

#### Orbicularis oculi

The statistical analyses showed no significant effect of either visual or psychological stress on orbicularis oculi blood flow. However, there was a main effect of time on muscle blood flow in the orbicularis oculi; *F*(1.74, 38.26) = 4.20, *p* = .027, *η*^2^ = 0.16, where overall muscle blood flow at 10 min was significantly higher than that during recovery; *F*(1, 22) = 7.93, *p* = .010, *η*^2^ = 27. (Fig. 3b).

### Muscle activity in the trapezius

**Fig. 4:**
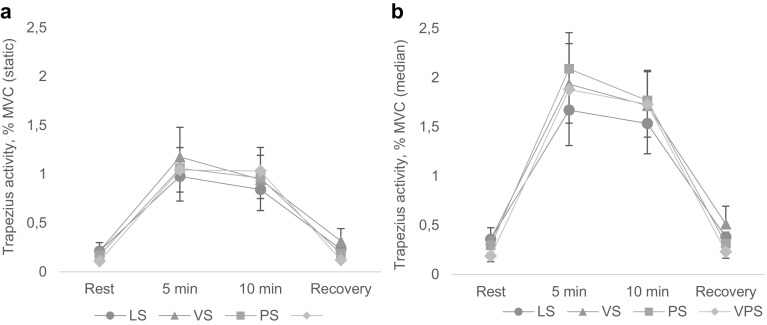
Muscle activity in trapezius: **a** static and **b** median load, during the four computer-work conditions. *LS* Low stress, *VS* visual stress, *PS* psychological stress, *VPS* psychological and visual stress. *Rest* = rest recording before computer work; *Recovery* = rest recording after a 14-min break (see Fig. 1). Results are given as mean ± SEM, *n* = 43

There was a borderline significant psychological stress by time interaction on trapezius muscle activity, median load; *F*(1.86, 77.95) = 3.13, *p* = .053, *η*^2^ = 07. During exposure to psychological stress, the results indicated that muscle activity at 5 min was significantly higher compared to that during rest; *F*(1, 42) = 4.85, *p* = .033, *η*^2^ = 0.10 (Fig. 4b). Muscle activity at 10 min and during recovery was not significantly different from that during rest, indicating an initial and transient increase in trapezius activity due to psychological stress.

In addition, Fig. 4 shows that trapezius muscle activity was higher during computer work compared to rest. The results showed a main effect of time on both static; *F*(1.17, 49.33) = 29.67, *p* < .001, *η*^2^ = 0.41, and median load; *F*(1.38, 57.80) = 48.86, *p* < .001, *η*^2^ = 54. This results revealed that overall muscle activity (median load) at 5 min; *F*(1, 42) = 32.10, *p* < .001, *η*^2^ = 56, and at 10 min; *F*(1, 42) = 30.12, *p* < .001, *η*^2^ = 57, was significantly higher than that during rest. Muscle activity during recovery was overall not significantly different from that during rest.

### Heart rate

**Fig. 5:**
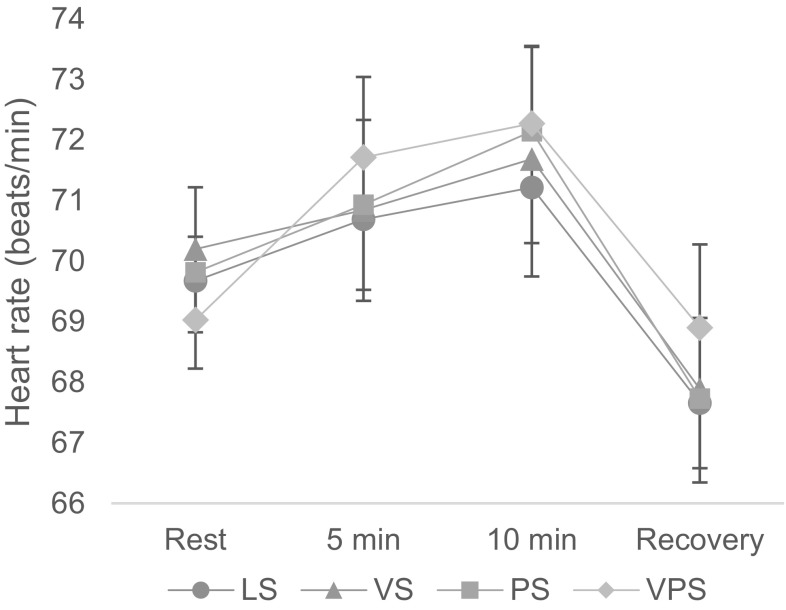
Heart rate during the four computer-work conditions. *LS* Low stress, *VS* visual stress, *PS* psychological stress, *VPS* psychological and visual stress. *Rest* = rest recording before computer work; *Recovery* = rest recording after a 14-min break (see Fig. 1). Results are given as mean ± SEM, *n* = 35

The results revealed no statistical significant effect of either visual or psychological stress on heart rate. However, Fig. 5 shows that there was a main effect of time on heart rate; *F*(1.27, 43.04) = 18.86, *p* < .001, *η*^2^ = 0.15. Overall, heart rate at 5 min; *F*(1, 34) = 6.08, *p* = .019, *η*^2^ = 0.36, and at 10 min; *F*(1, 34) = 10.62, *p* = .003, *η*^2^ = 0.24, was higher compared to that during rest, whereas heart rate during recovery; *F*(1, 34) = 40.57, *p* < .001, *η*^2^ = 0.54, was significantly lower than that during rest.

### Blood pressure

There were no statistical significant effect of exposure to visual or psychological stress on blood pressure (systolic and diastolic) during computer work. In average, the participants blood pressure during the computer-work periods was 106 ± 1 mm Hg (systolic) and 73 ± 1 mm Hg (diastolic) (mean ± SEM, *n* = 43).

### Work performance

Exposure to visual and psychological stress did not affect work performance, and there was no difference between conditions. Mean reading speed (productivity) in all conditions was 1691 ± 64 words per 10 min (mean ± SEM, *n* = 43), whereas mean accuracy was 84 ± 8% correctly marked errors in the text (mean ± SEM, *n* = 42).

### Postural angles

#### Back angles

**Fig. 6:**
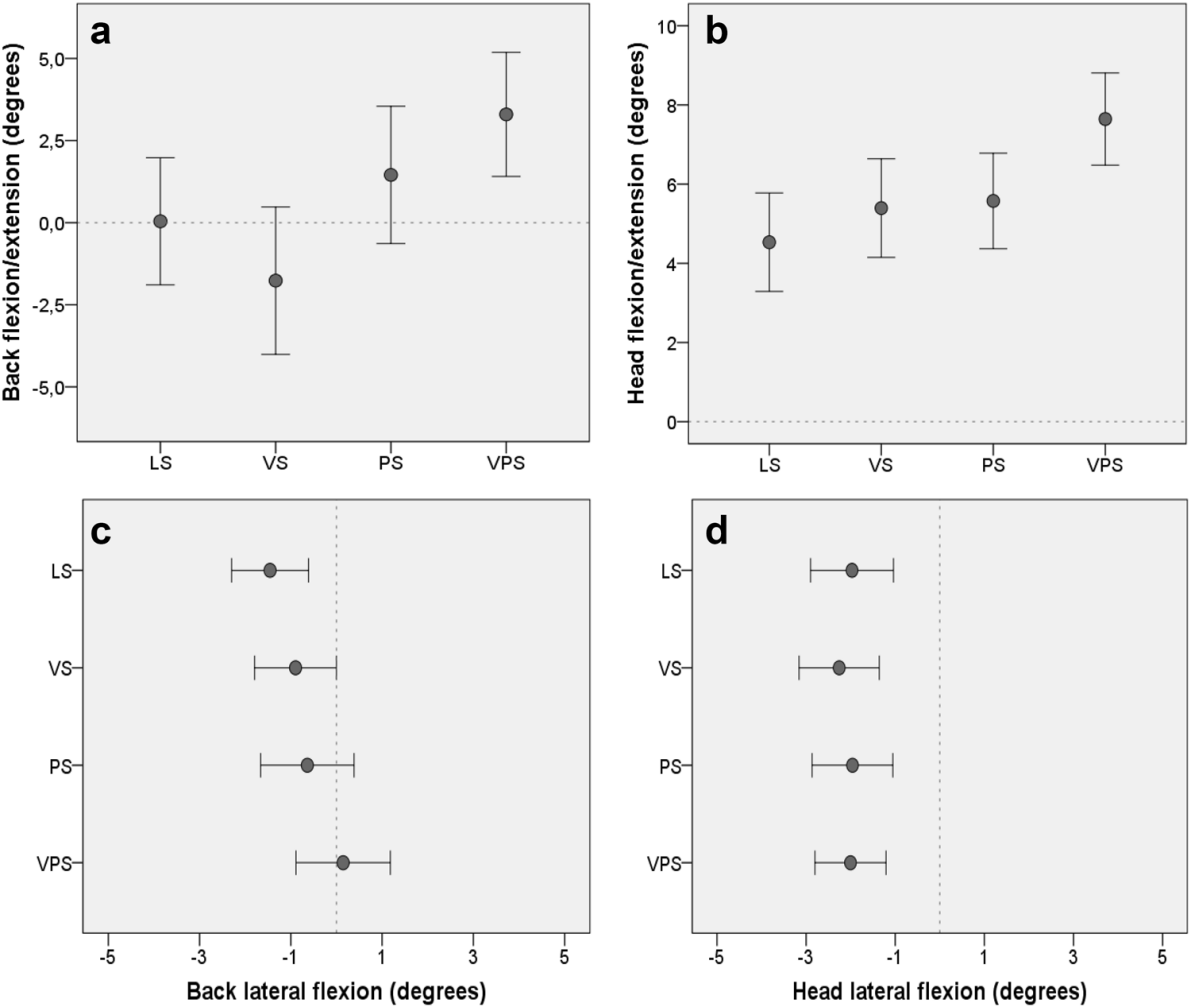
Postural angles for **a** back flexion/extension (leaning forward/backward, *n* = 35), **b** head flexion/extension (*n* = 38), **c** back lateral flexion (side bending, *n* = 31), and **d** head lateral flexion (*n* = 38) during the four computer-work conditions. *LS* Low stress, *VS* visual stress, *PS* psychological stress, *VPS* psychological and visual stress. Postural angles are given as degrees relative to a reference sitting position marked in the graphs with a dotted line, representing zero degrees (see “Methods”). Positive/negative values refer to forward/backward movements for flexion/extension and right/left movements for lateral flexion. Results are given as mean ± SEM

The analysis revealed a psychological stress by time interaction on back posture during computer work; *F*(2.17, 73.79) = 4.61, *p* = .011, *η*^2^ = 0.12, indicating that the participants moved closer to the computer screen by flexing their back during the induced psychological stress (Fig. 6a). When participants were exposed to psychological stress, their back was more forward bent at 5 min; *F*(1, 34) = 6.28, *p* = .017, *η*^2^ = 0.16, and at 10 min; *F*(1, 34) = 9.67, *p* = .004, *η*^2^ = 0.22, than during rest. There was no significant difference in back position between rest and recovery. Figure 6c shows that there was no significant effect on back lateral flexion of exposure to either visual or psychological stress during computer work.

#### Head angles

Figure 6b shows that both visual and psychological stress induced a more forward-bent head during computer work. The results showed both a statistically significant glare by time interaction; *F*(2.09, 77.38) = 6.19, *p* = .003, *η*^2^ = 0.14, and a psychological stress by time interaction; *F*(1.76, 65.12) = 3.47, *p* = .043, *η*^2^ = 0.09, on head flexion/extension. When participants were exposed to visual stress, their head was more forward bent at 5 min; *F*(1, 37) = 11.95, *p* = .001, *η*^2^ = 0.24, and at 10 min; *F*(1, 37) = 28.44, *p* < .001, *η*^2^ = 0.44, than during rest. When exposed to psychological stress, their head position at 5 min; *F*(1, 37) = 18.50, *p* < .001, *η*^2^ = 0.33, and at 10 min; *F*(1, 37) = 11.44, *p* = .002, *η*^2^ = 0.23, was also more forward bent than during rest. There was no significant difference in head flexion between rest and recovery. Figure 6d shows that there was no effect of either visual or psychological stress exposure on head lateral flexion during computer work.

#### Time effects on back and head angles

The results showed significant main effects of time on both back flexion/extension; *F*(1.56, 39.26) = 30.71, *p* < .001, *η*^2^ = 0.46, and head flexion/extension; *F*(1.61, 59.52) = 235.37, *p* < .001, *η*^2^ = 0.86. Compared to rest, the back and head were overall more forward bent at 5 min and at 10 min (*p* < .001), whereas they were more extended during recovery (*p* < .001). The results also showed a significant main effect of time on head lateral flexion; *F*(1.99, 73.69) = 7.49, *p* = .001, *η*^2^ = 0.17. The participants bent their head more leftward during computer work at 5 min; *F*(1, 37) = 9.81, *p* = .003, *η*^2^ = 0.21, and at 10 min; *F*(1, 37) = 8.40, *p* = .006, *η*^2^ = 0.19, compared to rest, whereas there was no difference in head lateral flexion between rest and recovery.

### Blink rate

**Fig. 7:**
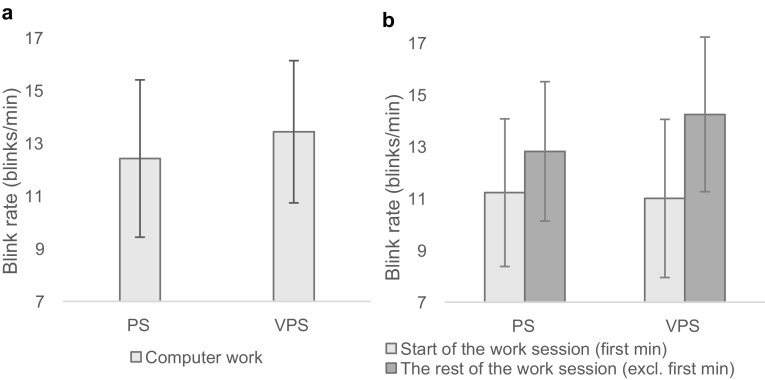
Blink rate in the two conditions with psychological stress exposure: **a** mean blink rate during the two computer-work sessions, and **b** mean blink rate at *start of the work session* (blinks per minute during 0–1 min) and at *the rest of the work session* (blinks per minute during the computer-work session, excluding the first minute). *PS* Psychological stress, *VPS* psychological and visual stress. Results are given as mean ± SEM, *n* = 18

Figure 7a shows that the participants blinked more frequently during the condition in which glare exposure was added to psychological stress, than they did during the condition with psychological stress only, indicating a blink response due to glare. The analysis revealed that there was a statistically significant main effect of condition on blink rate; *F*(1.00, 17.00) = 4.99, *p* = .039, *η*^2^ = 0.23.

In addition, Fig. 7b shows the blink rate at the beginning (blinks/min during first minute) and further during the computer-work sessions (average blinks/min during 2–9 min). The analysis revealed that there was a statistically significant effect of time on blink rate; *F*(1.00, 17.00) = 9.13, *p* = .008, *η*^2^ = 0.35, indicating that the participants increased their blink rate during later phases of the computer-work conditions.

### Fixation disparity

**Fig. 8:**
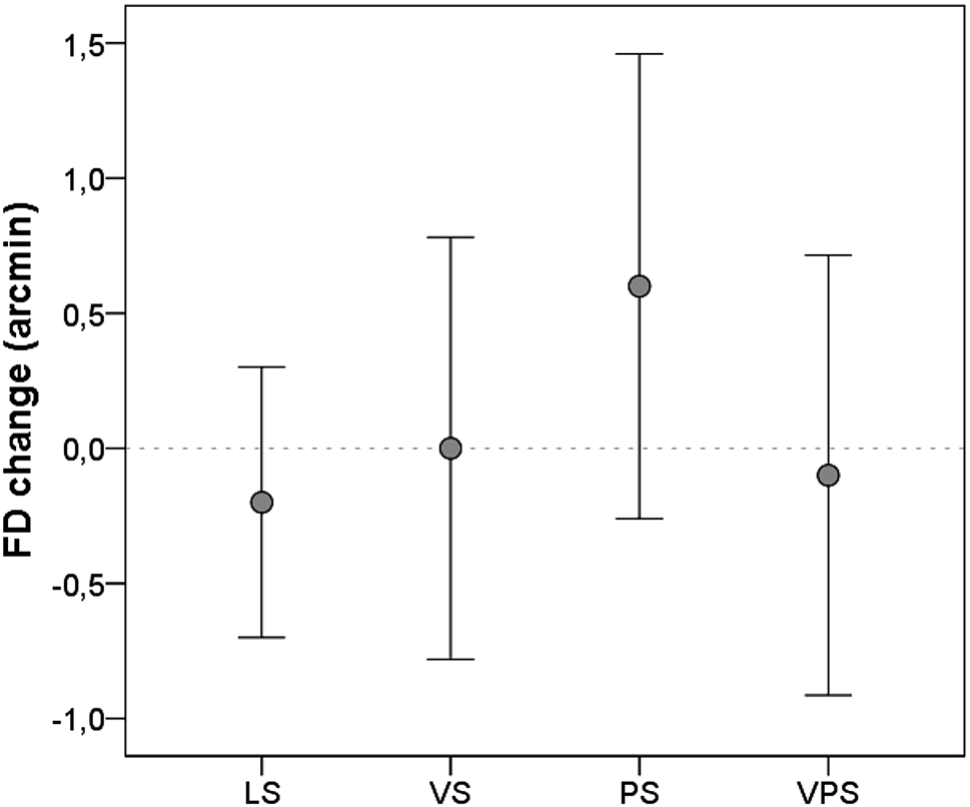
Mean FDchange for each of the computer-work conditions. *LS* Low stress, *VS* visual stress, *PS* psychological stress, *VPS* visual and psychological stress. Negative/positive values on y-axis represent a change in fixation disparity from baseline towards an exo-/eso-disparity. Dotted line represents the mean baseline value (zero). The results are given as mean ± SEM, *n* = 20

The mean fixation disparity was an exo-disparity at each measurement point (the eyes focus slightly behind the plane of focus). The mean fixation disparity was: at baseline: − 1.60 ± 0.99 (mean arc min ± SD, *n* = 20), after LS: − 1.80 ± 0.88, after VS: − 1.60 ± 1.05, after PS: − 1.00 ± 0.88, and after VPS: − 1.70 ± 1.10. Figure 8 shows results for FD_change_, the change in fixation disparity relative to baseline for each condition. There was no significant effect of visual or psychological stress on the fixation disparity measurements.

### Back angle associations

**Fig. 9:**
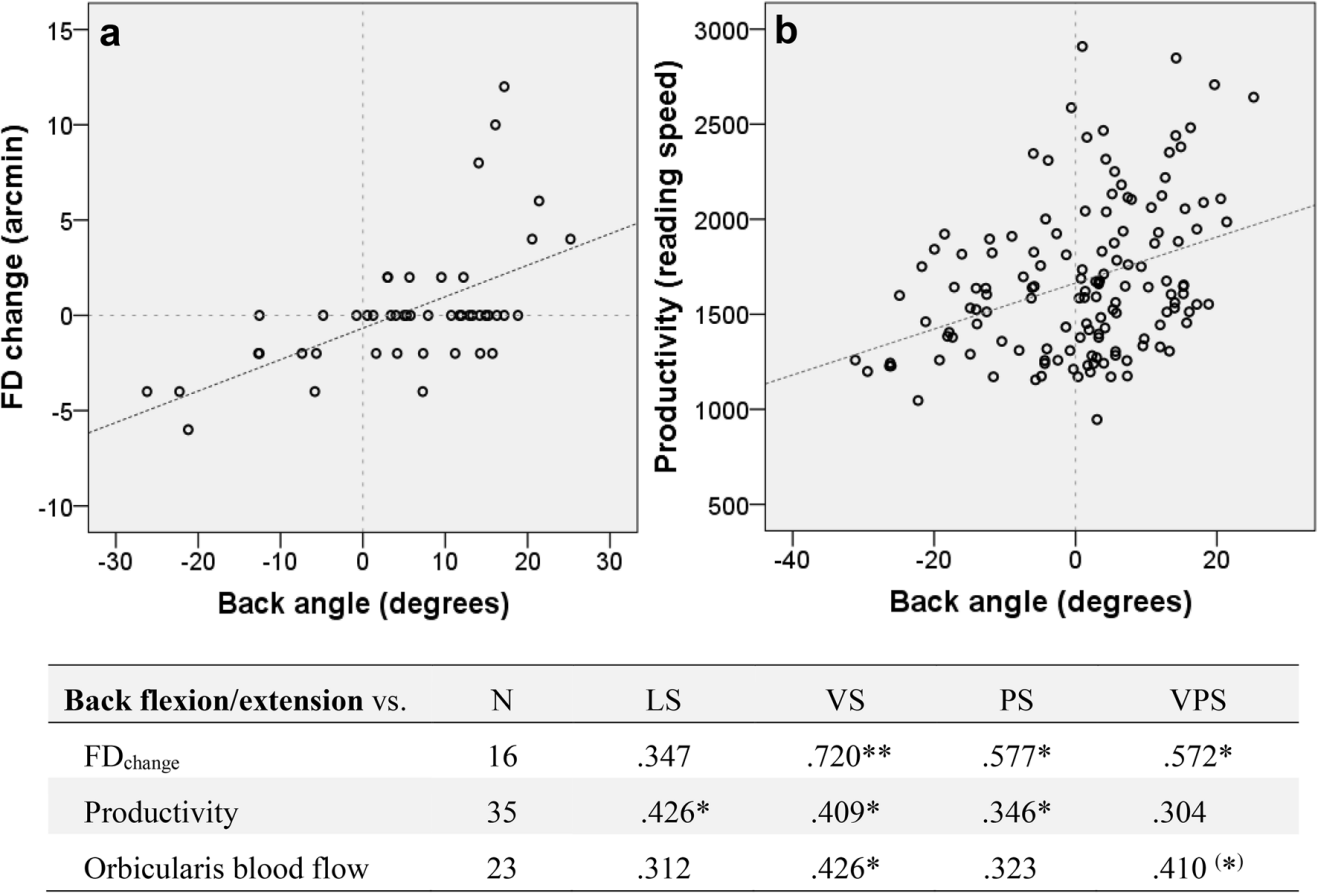
Significant correlations between back angle (leaning forward/backward = positive/negative values) and FDchange (change towards eso/exo-disparity = positive/negative values), productivity (reading speed, words/10 min), and orbicularis oculi muscle blood flow (in table). The correlations are given as Pearson’s correlation coefficients. The correlations plots show the associations between back angle and **a** FDchange in VS, PS and VPS, and **b** reading speed in LS, VS and PS. *LS* Low stress, *VS* visual stress, *PS* psychological stress, *VPS* visual and psychological stress. */**Statistically significant correlation at *p* < .05 and *p* < .01, respectively. (*) Close to, but not statistically significant correlation (*p* ≤ .060)

Figure 9 shows significant correlations between the participants’ back angle (flexion/extension) during computer work and FD_change_, reading speed, and orbicularis oculi blood flow. There were positive correlations between the participants’ back posture (leaning forward/backward) and both change in fixation disparity towards eso/exo-disparity relative to baseline (Fig. 9a) and higher/lower reading speed (Fig. 9b). In addition, there were positive correlations between back angle and orbicularis oculi blood flow during VS and VPS.

### Heart rate associations

Table 2 shows statistically significant correlations between heart rate and other measured parameters. In all conditions, increased heart rate was associated with reduced trapezius blood flow (or vice versa). Heart rate was also positively correlated with systolic blood pressure in LS and VPS and with perceived stress in PS and VPS. Further, there were negative associations between the participants’ heart rate and blink rate in VPS. This indicates that a rise in heart rate was accompanied with both reduced blink rate and trapezius blood flow, as well as with increased blood pressure and perceived stress during computer work.

**Table 2:** Summary of correlations between heart rate (beats/min) and trapezius muscle blood flow, systolic blood pressure (mmHg), perceived stress (mm VAS), and blink rate (blinks/min)

Heart rate vs.	*N*	LS	VS	PS	VPS
Trapezius blood flow	34	− 0.517**	− 0.404*	− 0.347*	− 0.338(*)
Systolic blood pressure	38	0.407*	0.204	0.293	0.352*
Stress	38	0.220	0.302	0.471**	0.350*
Blink rate, 1st min	18	–	–	− 0.307	− 0.530*
Blink rate, mean	18	–	–	− 0.295	− 0.492*

Results are given as Pearson’s correlation coefficients

*LS* Low stress, *VS* visual stress, *PS* psychological stress, *VPS* visual and psychological stress

*/**Statistically significant correlation at *p* < 0.05 and *p* < 0.01, respectively

(*) close to, but not statistically significant correlation (*p* ≤ .060)

### Overall effect of time during testing

For heart rate and head flexion/extension, there was a statistically significantly overall effect of time during testing. Heart rate decreased throughout testing from the first to the last condition, indicating that the participants were more stressed at the start of the test procedure than they were towards the end, independent of condition order. In addition, the head was also significantly more backward leaning in the third condition than in the first. Such overall effects of time might have washed out potentially significant effects.

## Discussion

In this study, young, healthy females with normal binocular vision were exposed to visual stress (direct glare) and psychological stress during computer work to elucidate the effects of these occupational simulated stressors. A common general stress response was expected both for visual and psychological stress. In addition, specific effects were expected during visual stress as an adaptation to glare.

### Effects of computer work per se

The participants’ trapezius muscle was affected by computer work as such, independent of exposure. This was shown by increased muscle activity and muscle blood flow while working compared to the rest period, and the most obvious explanation for this is active use of the trapezius while using the mouse to perform the computer task. Similar time effects in the trapezius were shown by Mork et al. (2016) during 30 min of reading on a computer. The recordings in Mork et al. (2016) also showed significant time effects on the trapezius muscle activity and muscle blood during glare exposure, but not during optimal lighting (similar to VS and LS in the current study, respectively). These measurements were performed on the non-dominant and passive trapezius as opposed to the dominant trapezius (active shoulder/arm) in the present study. This is an indication that factors other than active use of the trapezius also contribute to muscular changes associated with computer work in this neck and shoulder muscle.

Alterations in posture may also have contributed to the changes in the trapezius muscle. The participants’ sitting position was different during computer work compared to rest, and this could have affected the neck and shoulder musculature as a result of differences in postural load. However, no significant associations between postural angles and trapezius muscle activity or blood flow were present, which is consistent with Mork et al. (2016). This indicates that alterations in sitting position cannot solely explain the changes observed in trapezius during computer work. Westgaard and Bjørklund (1987) suggested that increased trapezius muscle activity during both mentally and visually demanding computer work could be induced by psychologically mediated muscle tension due to a variety of stressors associated with office work, unrelated to postural maintenance. In the current study, the task in all conditions was to read text on the computer screen and mark spelling errors. Reading is visually and cognitively demanding (Orchard and Stern 1991), and the verbal instructions before all conditions were to perform the task as well as possible. The task itself may therefore have placed an additional mental demand on the participants. During all the four computer-work periods, a general arousing effect due to the task may therefore have contributed to the increased trapezius muscle activity observed. Moreover, trapezius muscle blood flow is also previously reported to be affected by mental stress (Larsson et al. 1995). It is therefore likely that the increased trapezius muscle activity and muscle blood flow during computer work were affected by a similar mechanism. Further, the observed time effect of increased heart rate during computer work also supports that a general arousal was present during all conditions. In addition, heart rate was lowest during recovery, indicating that participants were more tense before start of the conditions compared to after completion.

In all conditions, there were negative associations between heart rate and trapezius muscle blood flow. In contrast, Larsson et al. (1995) found an increase in both heart rate and trapezius muscle blood flow due to mental stress. However, this mental stress was added to a series of static trapezius contractions (performed with straight arms elevated in different angles with a 1-kg load), inducing 30% muscle blood flow increase in the exercising trapezius. During low-force muscle activity, such as during the computer work in the present study, a different mechanism may be at work. Accordingly, neural control of muscle blood flow is shown to be complex (Shoemaker et al. 2016).

Further, the results showed that computer work itself also affected muscle blood flow in the orbicularis oculi, indicating that the orbicularis is activated during near-visual work and attention tasks. This has also been shown in previous research; computer work per se induce eyelid squinting and increased muscle blood flow in the orbicularis oculi (Mork et al. 2016). Squinting improves visual acuity and decreases the amount of light from the surroundings that enters the retina (Sheedy et al. 2003). In the present study, all participants had normal vision, and their orbicularis oculi response during computer work may therefore not have been caused by the need for improved vision/visual acuity; rather, it may have been an attention/concentration reflex.

The results corroborates previous research and elaborates further the magnitude and the pattern of effects connected to computer work. In the evolutionary stress model (Fostervold et al. 2014) the observed effects are interpreted as functional adaptations to demands imposed by near-visual work. Although work at close distances itself is not evolutionarily novel, prolonged static visual work at a short fixed distance, as in computer work, can be seen as an departure from the conditions from which the human visual system is adapted. Thus, it follows that continued efforts to cope with the situation, may ultimately give rise to secondary problems.

### Exposure to glare and psychological stress

As expected, the participants reported that they perceived worse workstation lighting in the glare conditions. The luminance of the visual object (computer screen) was 155 cd/m^2^, whereas the luminance of the glare source placed behind the computer screen was above 4500 cd/m^2^. Previous studies have reported that subjects prefer environmental luminance slightly below the luminance of the visual object (Sheedy et al. 2005), and ambient luminance above 600 cd/m^2^ has been rated as disturbing (Berman et al. 1994).

Participants rated perceived stress to be significantly higher in conditions with exposure to psychological stress. This was also expected, as stressors similar to those used in the current study previously have been shown to increase perceived stress levels in humans, as well as other psychological responses such as cortisol level and autonomic responses (Dickerson and Kemeny 2004; Skoluda et al. 2015; Wahlström et al. 2002). In the debriefing after the experiment, all participants confirmed that one or more of the induced psychological stressors had affected them, but there were intersubjective differences in what they reported to be the most stressful factor. The association between perceived stress and lighting in the condition with exposure to both glare and psychological stress, further indicates that poor ambient lighting may negatively influence perceived stress levels, or vice versa, during computer work.

#### Orbicularis oculi

There was no significant effect of either glare exposure or psychological stress on orbicularis oculi muscle blood flow in the present study. The low number of participants with complete orbicularis oculi blood flow data and the short exposure duration (10 min) may have influenced this result.

Mork et al. (2016) used the same glare source as in the present study, and reported glare to induce significantly increased orbicularis oculi muscle tension (eyelid squinting) during 30 min of computer reading (*n* = 15). In addition, a borderline significantly increased orbicularis oculi blood flow was observed. The eyelid squinting response may reflect an adaptation of the orbicularis muscle in the presence of glare, to reduce the amount of light entering the eyes (Sheedy et al. 2003). Accordingly, it is likely to anticipate increased muscle activity in orbicularis oculi also in the present study. Further, the results revealed that during glare exposure, orbicularis oculi muscle blood flow was affected by sitting posture. Bending forward towards the screen/glare source was associated with increased muscle blood flow. This may be related to a stronger eyelid squinting response due to higher retinal illumination as the distance to the glare source became shorter. In relation to this, Thorud et al. (2012) have previously shown that both muscle blood flow and muscle activity in orbicularis oculi is related to increased eye-related symptoms.

The hypothesis for a psychological stress response in orbicularis oculi was outlined based on that psychological stress has previously been shown to affect the hemodynamics and/or muscle activity of other facial muscles (Hidaka et al. 2004a, b; Nilsen et al. 2007). The current study did not find any effect of psychological stress on orbicularis oculi muscle blood flow, indicating that blood flow in this muscle may not be affected by psychological stress. However, orbicularis oculi muscle activity was not recorded in this study.

#### Cardiovascular responses

In the present study, either glare or psychological stress did significantly affect the cardiovascular responses in the participants. The literature is inconclusive regarding heart rate and mental loads, showing both increased heart rate and no effect due to psychological stress exposure (Hidaka et al. 2004b; Iwanaga et al. 2000; Larsson et al. 1995; Nahar et al. 2011; Skoluda et al. 2015). Skoluda et al. (2015) found that the laboratory stress protocol Trier Social Stress Test (TSST), which includes a social-evaluation threat and a mental task, induced a perceived stress level of 30 mm VAS in young men. TSST also induced increased heart rate and alpha-amylase concentration (indicating increased sympathetic activity) and increased cortisol concentration in saliva (indicating activation of a stress response in the hypothalamus–pituitary–adrenal axis). The psychological stress conditions in the present study had some similarities to the study of Skoluda et al. (2015): it included social-evaluation threat exposure, the conditions had similar duration, and the participants reported perceived stress at approximately the same level (30–35 mm VAS). Together with the discrepancy according the effect of mental stress on heart rate, this may indicate that even though the effect on heart rate was not statistically significant, the psychological exposure might have evoked other responses in the participants. In line with this, we did find a positive correlation between heart rate and perceived stress in the conditions with psychological stress exposure.

Another explanation for the lack of cardiovascular responses in the current study is that the psychological stressors may have been too weak. Hidaka et al. (2004b) observed an increase in sympathetic nervous activity when exposing females to a weak and long-lasting mental stressor, but there was no accompanying heart rate increase even though the stress was reported as “extremely stressful” (4 on a 0–4 point scale). This may further indicate a possibility for the psychological stress in the present study to provoke a sympathetic response in the participants, but without an accompanying increase in heart rate.

The literature on the effect of persistent glare exposure on heart rate in human is limited (except from effects of bright light/glare in depressed patients, night shift-workers and drivers) (Mork et al. 2016; Saito et al. 1996). However, the absence of a glare effect on heart rate indicates that the set up in the current study was not able to provoke a heart rate response in young, healthy females.

#### Blink rate

Blink rate was significantly affected by glare exposure in the current study. This is in accordance with earlier studies showing that glare (and refractive error) induces more frequent blinks (Gowrisankaran et al. 2007; Nahar et al. 2011, 2007). Increased blink rate is understood as an indication of ocular fatigue or discomfort (Rodriguez et al. 2018; Stern et al. 1994), which suggests that the increased blink rate observed during glare exposure, may be due to fatigue/discomfort caused by additional stress placed on the visual system.

Further, the participants’ blink rate was less frequent during the first minute compared to later phases of the computer-work conditions, and this occurred simultaneously with an initial and transient increase in trapezius muscle activity. This indicated that the participants were most concentrated or stressed in the first part of the computer-work periods, which is consistent with studies showing that reduced blink rate is an aspect of a stress/concentration response (Gowrisankaran et al. 2012; Iwanaga et al. 2000; Rosenfield et al. 2015). The significant negative relationship shown between heart rate and blink rate also supports this view. In addition, muscle activation in the trapezius is also considered an indicator of mental stress (Lundberg et al. 2002; Westgaard 1999).

The observed pattern of findings seem to concur both with previous findings showing reduced blink frequency in computer work (Nielsen et al. 2008; Wolkoff 2008) and predictions made by the evolutionary stress model (Fostervold et al. 2014). Near-work are generally concentration intensive and from an evolutionary viewpoint, it seems adaptive to reduce the blink frequency to maintain focus. However, the intensity and duration of near-work found in computer work vastly exceeds the conditions for which the human visual system is adapted. Continued efforts to cope will gradually lead to desiccation of the precorneal tear film and dry eyes (Nielsen et al. 2008; Wolkoff 2008). Dry eyes and unstable tear film seems to increase scattering and dispersion of light in the eye and contributes to reduced retinal image contrast and increased risk of disability glare (Huang et al. 2002; Puell et al. 2006). As the main objective of the visual system is to maintain sharp and single images at the retina, increased blink frequency seems thus to be an adaptive response to the demands imposed by the environment.

#### The trapezius muscle

The trapezius muscle was affected by both glare and psychological stress in the current study. During glare, the trapezius muscle blood flow increased significantly, whereas the trapezius muscle activity increased initially due to psychological stress, suggesting that the visual and psychological stress responses were dissimilar. Different stimuli that act as stressors evoke a wide range of physiological responses, including a variety of autonomic, hormonal, and behavioural responses (Dampney 2015; Dayas et al. 2001). In line with this, visual stressors and psychosocial stressors have different complex pathways into the brain (e.g., into the dorsolateral periaqueductal grey which has an important role in the coordination of responses to a variety of stressors) (Bergmanson 2017; Chellappa et al. 2017; Dampney 2015; Dedovic et al. 2009). The dissimilar responses observed regarding trapezius muscle blood flow and muscle activity is therefore not surprising.

The significant increase in trapezius muscle blood flow during glare exposure was not present during computer work with appropriate lighting. This implies the presence of a specific visual stress response due to glare, as previously shown (Mork et al. 2016). Glare is potentially detrimental for functional vision and specific responses to glare was therefore expected. Whether or not the observed response in trapezius represents a functional adaptation in evolutionary terms is difficult to say for sure, as its functional and neurological basis is still unclear. However, some possible explanations can be outlined.

Glare may have produced a response in the autonomic sympathetic nervous system (Belkić 1986; Saito et al. 1996). Saito et al. (1996) found increased activity in the sympathetic nervous system during exposure to excessive light (5000 lx) for 20 min, and activation of autonomic responses is known to affect muscle blood flow in skeletal muscles (Shoemaker et al. 2016). It has also been shown that vasodilatation in the muscles of cats occurs in the early, alerting stage of the defense reaction, produced both by direct electrical stimulation of the hypothalamus and by environmental stimuli, such as flash of light (Abrahams et al. 1964). The optic nerve in humans leads visual input directly into important cortical regions regulating autonomic responses, such as the hypothalamus (Royet et al. 2000), and an activation of the autonomic nervous system due to visual stress may therefore be a possible explanation for the increased blood flow observed in the trapezius muscle during glare in the current study.

Further, glare/excessive stray light on the retina leads to reduced contrast, altered visibility, and blur (Fry and Alpern 1953; Lie 1981; Van Den Berg 1991). Both blur and excessive light exposure has been shown to affect eye-lens accommodation (Kruger and Pola 1986; Shahnavaz and Hedman 1984; Wolska and Switula 1999). In line with this, Richter and coworkers have in several studies shown associations between accommodation/ciliary muscle contraction and a bilateral increase in trapezius activity (Richter et al. 2010; Richter and Forsman 2011; Zetterberg et al. 2013). They stated that this interaction between the eyes and the neck/shoulder muscle may be a neural command between sustained eye-lens accommodation when fixating on a near target and the postural muscles in the neck and shoulder area, which is activated to stabilize gaze during visually demanding conditions, such as near-visual work. In line with this, proprioceptive information from the oculomotor muscles and from somatic neck muscles has been shown to be mutually influential (Biguer et al. 1982; Bruenech et al. 2012; Han and Lennerstrand 1995, 1998). The eyes and the head are activated synchronously with eye movements when looking at a visual target (Biguer et al. 1982), and proprioceptive signals (vibration) of different neck muscles induce eye movements (Han and Lennerstrand 1995). This implies that proprioceptive messages originating in the eye muscles and/or the neck muscles are processed together and lead to coordinated activation. In line with this, relationships between eyestrain and eyelid squinting, and neck and shoulder muscles and musculoskeletal symptoms have been shown (Helland et al. 2008; Mork et al. 2016; Richter et al. 2011; Wiholm et al. 2007; Zetterlund et al. 2009). To the best knowledge of the authors, no study has investigated associations between trapezius muscle blood flow and accommodation or oculomotor muscles. Therefore, a relationship between ocular muscles and neck muscles cannot be dismissed as a factor involved in the increased muscle blood flow in trapezius during glare exposure.

The observed trapezius blood flow increase could also be influenced by changes in posture affecting muscles in the neck and shoulder region. However, there were no differences in postural angles in the conditions with glare compared to the no-glare conditions. In fact, the back angle was opposite in the two conditions with glare: extended during the condition with glare only and flexed during the condition with both glare and psychological stress. Further, there were no significant associations between trapezius blood flow and the postural angles measured. Relationships have been found between rotation of the head away from the midline and neck and shoulder complaints and pain (Faucett and Rempel 1994). Head rotation was not registered in the present study, and therefore the possibility that head rotation affects the trapezius muscle cannot be excluded. However, Fig. 6 shows that the participants exhibited similar movement patterns for both head flexion and head lateral flexion in all four conditions and that the differences in angle between the conditions was small (within 2°–3°). It is therefore unlikely that head rotation is the explanation for the observed blood flow increase during glare exposure.

#### Posture; associations to concentration and vision

The participants’ posture was affected by the different induced stress exposures during computer work in the present study. Glare made the participants bend their head forward, probably to keep excessive light from entering the eyes. Psychological stress made the participants bend both their head and back significantly more forward, and a plausible explanation for this is that mental work/demanding concentration tasks induce a more forward bent position to increase proximity to the visual object of interest. The finding that the participants were significantly more productive when bent forward also supports the suggestion that leaning forward is a concentration response.

Fixation disparity measured as exo-disparities was expected, because all measurements were captured at near distances (Jaschinski 2017). However, we did not find any significant effect on fixation disparity attributable to the visual stress, and this contrasts with other findings. Glimne et al. (2013) showed significantly more variation in fixation disparity after computer work with glare exposure compared to non-glare conditions. However, they used a fixed chin and forehead rest to control for the viewing distance during the experiment. In the present study, participants were allowed to alter their posture and move freely during computer work. There were significantly positive associations between back angle and change in fixation disparity relative to baseline in the conditions with visual and/or psychological stress. This indicate that participants compensated for alterations in fixation disparity by moving closer to or farther away from the computer screen when exposed to additional stress. This seems reasonable, because a longer or shorter viewing distance releases the outward (exo) or inward (eso) demand on the binocular system, respectively. This is also supported by Jaschinski (2002), who reported that when able to choose a comfortable viewing distance during computer work, subjects preferring longer viewing distances had more exo-disparity during near-visual work (with forced distance) than did subjects preferring shorter distances. These results indicate that visual factors may affect working posture, or vice versa, during computer work with exposure to additional environmental stressors. Either way, this suggests that persistent exposure to visual and/or psychological stress can lead to visual and musculoskeletal complaints and reduced work capacity due to altered demand on the binocular system or altered postural load. Further research is needed to understand these connections, but the results highlights the necessity for optimized visual ergonomics during computer work.

## Conclusion

Exposure to visual and psychological stress during computer work affects young, healthy females with normal binocular vision, but the stress mechanisms are obviously dissimilar.

During visual stress (direct glare), the trapezius muscle blood flow increased and the participants bent their head forward, probably to reduce the amount of light entering the retina. Glare exposure also resulted in increased blink rate, possibly as an adaptation due to ocular discomfort. Psychological stress induced a transient increase in trapezius muscle activity at the start of the computer-work sessions, as well as affecting the sitting position by resulting in a more forward-bent posture. Bending forward towards the computer screen was associated with higher reading speed, indicating a concentration or stress response. Forward bent posture was also associated with changes in fixation disparity. Furthermore, during computer work per se, trapezius muscle activity and muscle blood flow, orbicularis oculi muscle blood flow, and heart rate were increased compared to rest.

This study emphasizes that problems and ailments associated with computer work should be understood in a broad environmental context. The traditional medical and biomechanical model is often restricted to discussing proximate relationships (i.e., questions concerning how the body works and why some people develop symptoms and diseases). The evolutionary approach extend this analytical limitation further by encompassing questions about why some body parts are developed to tolerate high amounts of strain, while others develop malfunctions in their analysis of health problems (Scott-Phillips et al. 2011).

When adjusting computer workplaces, it is important to not only minimize potential environmental stress, but also to understand why some situations are tolerated quite well, while others seem to put undue strain upon the visual system. This study shows that the optimizing of computer workstations is a complex field that must take into account several different factors, including both physical and psychological factors. This requires a multidisciplinary approach and visual ergonomics should be included, because visual conditions affect the worker during computer work.

## References

[CR1] Aaras A, Ro O (1997). Electromyography (EMG)—methodology and application in occupational health. Int J Ind Ergon.

[CR2] Aaras A, Stranden E (1988). Measurement of postural angles during work. Ergonomics.

[CR3] Aaras A, Veierød M, Larsen S, Ortengren R, Ro O (1996). Reproducibility and stability of normalized EMG measurements on musculus trapezius. Ergonomics.

[CR4] Abrahams VC, Hilton SM, Zbrożyna AW (1964). The role of active muscle vasodilation in the alerting stage of the defence reaction. J Physiol.

[CR5] Andersen JH, Fallentin N, Thomsen JF, Mikkelsen S (2011). Risk factors for neck and upper extremity disorders among computers users and the effect of interventions: an overview of systematic reviews. PLOS One.

[CR6] Anshel JR (2007). Visual ergonomics in the workplace. AAOHN J.

[CR7] Arbeidsplassforskriften (2011) Forskrift om utforming og innretning av arbeidsplasser og arbeidslokaler (norwegian). Work place guidelines, Chap 2. Norway. http://lovdata.no/dokument/SF/forskrift/2011-12-06-1356/KAPITTEL_2#§2-16

[CR8] Belkić K (1986). Light stress and the cardiovascular system: the glare pressor test. Ergonomics.

[CR9] Bergmanson JPG (2017). Clinical ocular anatomy and physiology.

[CR10] Berman SM, Bullimore MA, Jacobs RJ, Bailey IL, Gandhi N (1994). An objective measure of discomfort glare. J Illum Eng Soc.

[CR11] Biguer B, Jeannerod M, Prablanc C (1982). The coordination of eye, head, and arm movements during reaching at a single visual target. Exp Brain Res.

[CR12] Bizzi E, Kalil RE, Tagliasco V (1971). Eye-head coordination in monkeys: evidence for centrally patterned organization. Science.

[CR13] Boyce P (2014). Human factors in lighting.

[CR14] Bron AJ, Tripathi RC, Tripathi BJ (1997). Wolff’s anatomy of the eye and orbit.

[CR15] Bruenech JR, Kjellevold Haugen I-B, Bak U, Maagaard M, VanderWerf F (2012). The oculomotor systems ability to adapt to structural changes caused by the process of Senescence: a review. SJOVS.

[CR16] Chellappa SL, Lasauskaite R, Cajochen C (2017). In a heartbeat: light and cardiovascular physiology. Front Neurol.

[CR17] Collins A, Frankenhaeuser M (1978). Stress responses in male and female engineering students. J Hum Stress.

[CR18] Dampney RAL (2015). Central mechanisms regulating coordinated cardiovascular and respiratory function during stress and arousal. Am J Physiol.

[CR19] Dayas CV, Buller KM, Crane JW, Xu Y, Day TA (2001). Stressor categorization: acute physical and psychological stressors elicit distinctive recruitment patterns in the amygdala and in medullary noradrenergic cell groups. Eur J Neurosci.

[CR20] Dedovic K, D’Aguiar C, Pruessner JC (2009). What stress does to your brain: a review of neuroimaging studies. Canadian J Psychiatry.

[CR21] Dickerson S, Kemeny M (2004). Acute stressors and cortisol responses: a theoretical integration and synthesis of laboratory research. Psychol Bull.

[CR22] Dwyer PS (1982). Instrument review: the sheedy fixation disparometer. Aust J Optometry.

[CR23] Ellis CJ (1981). The pupillary light reflex in normal subjects. Br J Ophthalmol.

[CR24] Faucett J, Rempel D (1994). VDT-related musculoskeletal symptoms: interactions between work posture and psychosocial work factors. Am J Ind Med.

[CR25] Fostervold KI (2003). VDU work with downward gaze: the emperor’s new clothes or scientifically sound?. Int J Ind Ergon.

[CR26] Fostervold KI, Aarås A, Lie I (2006). Work with visual display units: long-term health effects of high and downward line-of-sight in ordinary office environments. Int J Ind Ergon.

[CR27] Fostervold K, Watten R, Volden F (2014). Evolutionary adaptations: theoretical and practical implications for visual ergonomics. Work (Reading Mass).

[CR28] Fry GA, Alpern M (1953). The effect of a peripheral glare source upon the apparent brightness of an object. J Opt Soc Am.

[CR29] Gerr F (2002). A prospective study of computer users: I. Study design and incidence of musculoskeletal symptoms and disorders. Am J Ind Med.

[CR30] Girardi D, Falco A, Dal Corso L, Kravina L (2011). Interpersonal conflict and perceived work stress: the role of negative affectivity. TPM.

[CR31] Glimne S, Seimyr G, Ygge J, Nylén P, Brautaset RL (2013). Measuring glare induced visual fatigue by fixation disparity variation. Work.

[CR32] Glimne S, Brautaset RL, Seimyr GO (2015). The effect of glare on eye movements when reading. Work.

[CR33] Gowrisankaran S, Sheedy JE (2015). Computer vision syndrome: a review. Work.

[CR34] Gowrisankaran S, Sheedy JE, Hayes JR (2007). Eyelid squint response to asthenopia-inducing conditions. Optom Vis Sci.

[CR35] Gowrisankaran S, Nahar NK, Hayes JR, Sheedy JE (2012). Asthenopia and blink rate under visual and cognitive loads. Optom Vis Sci.

[CR36] Han Y, Lennerstrand G (1995). Eye movements in normal subjects induced by vibratory activation of neck muscle proprioceptors. Acta Ophthalmol Scand.

[CR37] Han Y, Lennerstrand G (1998). Effects of neck muscle proprioceptive activation on the dynamics of monocularly driven horizontal vergence movements. Acta Ophthalmol Scand.

[CR38] Helland M, Horgen G, Kvikstad TM, Garthus T, Bruenech JR, Aaras A (2008). Musculoskeletal, visual and psychosocial stress in VDU operators after moving to an ergonomically designed office landscape. Appl Ergon.

[CR39] Hidaka O, Yanagi M, Takada K (2004). Changes in masseteric hemodynamics time-related to mental stress. J Dent Res.

[CR40] Hidaka O, Yanagi M, Takada K (2004). Mental stress-induced physiological changes in the human masseter muscle. J Dent Res.

[CR41] Huang F-C, Tseng S-H, Shih M-H, Chen FK (2002). Effect of artificial tears on corneal surface regularity, contrast sensitivity, and glare disability in dry eyes. Ophthalmology.

[CR42] ISO 9241-5 (1998) Ergonomic requirements for office work with visual display terminals (VDTs). Part 5: Workstation layout and postural requirements

[CR43] Iwanaga K, Saito S, Shimomura Y, Harada H, Katsuura T (2000). The effect of mental loads on muscle tension, blood pressure and blink rate. J Physiol Anthropol Appl Hum Sci.

[CR44] Jaschinski W (2002). The proximity-fixation-disparity curve and the preferred viewing distance at a visual display as an indicator of near vision fatigue. Optom Vis Sci.

[CR45] Jaschinski W (2017). Individual objective and subjective fixation disparity in near vision. PLoS One.

[CR46] Johnson G, Bogduk N, Nowitzke A, House D (1994). Anatomy and actions of the trapezius muscle. Clin Biomech.

[CR47] Jonsson B (1982). Measurement and evaluation of local muscular strain in the shoulder during constrained work. J Hum Ergol (Tokyo).

[CR48] Jun D, Zoe M, Johnston V, O’Leary S (2017). Physical risk factors for developing non-specific neck pain in office workers: a systematic review and meta-analysis. Int Arch Occup Environ Health.

[CR49] Kreibig S (2010). Autonomic nervous system activity in emotion: a review. Biol Psychol.

[CR50] Kruger PB, Pola J (1986). Stimuli for accommodation: blur, chromatic aberration and size. Vision Res.

[CR51] Larsson SE, Larsson R, Zhang Q, Cai H, Oberg PA (1995). Effects of psychophysiological stress on trapezius muscles blood flow and electromyography during static load. Eur J Appl Physiol Occup Physiol.

[CR52] Larsson B, Søgaard K, Rosendal L (2007). Work related neck–shoulder pain: a review on magnitude, risk factors, biochemical characteristics, clinical picture and preventive interventions. Best Pract Res Clin Rheumatol.

[CR53] Levin LA, Nilsson SFE, Kaufman PL, Alm A, Ver Hoeve J, Wu SM (2011). Adler’s physiology of the eye.

[CR54] Lie I (1981). Visual detection and resolution as a function of adaptation and glare. Vision Res.

[CR55] Lie I, Watten RG (1987). Oculomotor factors in the aetiology of occupational cervicobrachial diseases (OCD). Eur J Appl Physiol.

[CR56] Lie I, Watten RG (1994). VDT work, oculomotor strain, and subjective complaints: an experimental and clinical study. Ergonomics.

[CR57] Lie I, Watten R, Fostervold K, Frantzen O, Richter H, Stark L (2000). Accommodation/vergence/fixation disparity and synergism of head, neck and shoulders. accommodationivergence mechanisms in the visual system.

[CR58] Lillelien E, Skar JP, Fosse KM, Berg MO (2012). Lux-table and planning criteria for indoor lighting systems (norwegian). Lyskultur—norsk kunnskapssenter for lys.

[CR59] Lindberg LG, Oberg PA (1991). Photoplethysmography. Part 2. Influence of light source wavelength. Med Biol Eng Comput.

[CR60] Luine V, Gomez J, Beck K, Bowman R (2017). Sex differences in chronic stress effects on cognition in rodents. Pharmacol Biochem Behav.

[CR61] Lundberg U, Forsman M, Zachau G, Eklöf M, Palmerud G, Melin B, Kadefors R (2002). Effects of experimentally induced mental and physical stress on motor unit recruitment in the trapezius muscle. Work Stress.

[CR62] Mohanty P, Singh A, Pattnaik M (2017). Risk factors responsible for musculoskeletal pain among computer operators. EC Orthopaedics.

[CR63] Mork R, Bruenech JR, Thorud HMS (2016). Effect of direct glare on orbicularis oculi and trapezius during computer reading. Optom Vis Sci.

[CR64] Nahar NK, Sheedy JE, Hayes J, Tai Y-C (2007). Objective measurements of lower-level visual stress. Optom Vis Sci.

[CR65] Nahar NK, Gowrisankaran S, Hayes JR, Sheedy JE (2011). Interactions of visual and cognitive stress. Optometry.

[CR66] Neely JC (1956). The R.A.F. Near-point Rule. Br J Ophthalmol.

[CR67] Nielsen PK, Søgaard K, Skotte J, Wolkoff P (2008). Ocular surface area and human eye blink frequency during VDU work: the effect of monitor position and task. Eur J Appl Physiol.

[CR68] Nilsen KB, Sand T, Stovner LJ, Leistad RB, Westgaard RH (2007). Autonomic and muscular responses and recovery to one-hour laboratory mental stress in healthy subjects. BMC Musculoskelet Disord.

[CR69] Orchard LN, Stern JA (1991). Blinks as an index of cognitive activity during reading. Integr Physiol Behav Sci.

[CR70] Ortego G, Villafañe JH, Doménech-García V, Berjano P, Bertozzi L, Herrero P (2016). Is there a relationship between psychological stress or anxiety and chronic nonspecific neck-arm pain in adults? A systematic review and meta-analysis. J Psychosom Res.

[CR71] Owen DB (1962). Handbook of statistical tables.

[CR72] Paksaichol A, Janwantanakul P, Purepong N, Pensri P, van der Beek AJ (2012). Office workers’ risk factors for the development of non-specific neck pain: a systematic review of prospective cohort studies. Occup Environ Med.

[CR73] Pickwell LD, Yekta AA, Jenkins TC (1987). Effect of reading in low illumination on fixation disparity. Am J Optom Physiol Opt.

[CR74] Price DD, Bush FM, Long S, Harkins SW (1994). A comparison of pain measurement characteristics of mechanical visual analogue and simple numerical rating scales. Pain.

[CR75] Puell MC (2006). Contrast sensitivity and disability glare in patients with dry eye. Acta Ophthalmol Scand.

[CR76] Ranasinghe P, Wathurapatha WS, Perera YS, Lamabadusuriya DA, Kulatunga S, Jayawardana N, Katulanda P (2016). Computer vision syndrome among computer office workers in a developing country: an evaluation of prevalence and risk factors. BMC Res Notes.

[CR77] Richter HO, Forsman M (2011). Accommodation/vergence eye movements and neck/scapular muscular activation: gaze control with relevance for work-related musculoskeletal disorders. Curr Trends Neurol.

[CR78] Richter HO, Bänziger T, Abdi S, Forsman M (2010). Stabilization of gaze: a relationship between ciliary muscle contraction and trapezius muscle activity. Vision Res.

[CR79] Richter HO, Zetterlund C, Lundqvist L-O (2011). Eye-neck interactions triggered by visually deficient computer work. Work.

[CR80] Rodriguez JD, Lane KJ, Ousler GW, Angjeli E, Smith LM, Abelson MB (2018). Blink: characteristics, controls, and relation to dry eyes. Curr Eye Res.

[CR81] Rosenfield M (2011). Computer vision syndrome: a review of ocular causes and potential treatments. Ophthalmic Physiol Opt.

[CR82] Rosenfield M, Jahan S, Nunez K, Chan K (2015). Cognitive demand, digital screens and blink rate. Comput Hum Behav.

[CR83] Royet J-P, Zald D, Versace R, Costes N, Lavenne F, Koenig O, Gervais R (2000). Emotional responses to pleasant and unpleasant olfactory, visual, and auditory stimuli: a positron emission tomography study. J Neurosci.

[CR84] Saito Y (1996). Effect of bright light exposure on muscle sympathetic nerve activity in human. Neurosci Lett.

[CR85] Sandberg M, Zhang Q, Styf J, Gerdle B, Lindberg LG (2005). Non-invasive monitoring of muscle blood perfusion by photoplethysmography: evaluation of a new application. Acta Physiol Scand.

[CR86] Scott-Phillips TC, Dickins TE, West SA (2011). Evolutionary theory and the ultimate–proximate distinction in the human behavioral sciences. Perspect Psychol Sci.

[CR87] Shahnavaz H, Hedman L (1984). Visual accommodation changes in VDU-operators related to environmental lighting and screen quality. Ergonomics.

[CR88] Sheedy JE, Truong SD, Hayes JR (2003). What are the visual benefits of eyelid squinting?. Optometry Vision Sci.

[CR89] Sheedy JE, Smith R, Hayes J (2005). Visual effects of the luminance surrounding a computer display. Ergonomics.

[CR90] Shoemaker JK, Badrov MB, Al-Khazraji BK, Jackson DN (2016). Neural control of vascular function in skeletal muscle. Comprehensive Physiol.

[CR91] Skoluda N (2015). Intra-individual psychological and physiological responses to acute laboratory stressors of different intensity. Psychoneuroendocrinology.

[CR92] Stern JA, Boyer D, Schroeder D (1994). Blink rate: a possible measure of fatigue. Hum Factors.

[CR93] Thorud HM, Helland M, Aaras A, Kvikstad TM, Lindberg LG, Horgen G (2012). Eye-related pain induced by visually demanding computer work. Optom Vis Sci.

[CR94] Van Den Berg TJTP (1991). On the relation between glare and straylight. Doc Ophthalmol.

[CR95] Wærsted M, Hanvold TN, Veiersted KB (2010). Computer work and musculoskeletal disorders of the neck and upper extremity: a systematic review. BMC Musculoskelet Disord.

[CR96] Wahlström J, Hagberg M, Johnson P, Svensson J, Rempel D (2002). Influence of time pressure and verbal provocation on physiological and psychological reactions during work with a computer mouse. Eur J Appl Physiol.

[CR97] Watson D, Clark LA, Tellegen A (1988). Development and validation of brief measures of positive and negative affect: the PANAS scales. J Pers Soc Psychol.

[CR98] Westgaard RH (1999). Effects of physical and mental stressors on muscle pain. Scand J Work Environ Health.

[CR99] Westgaard RH, Bjørklund R (1987). Generation of muscle tension additional to postural muscle load. Ergonomics.

[CR100] Wiholm C, Richter H, Mathiassen SE, Toomingas A (2007). Associations between Eyestrain and neck-shoulder symptoms among call-center operators. SJWEH Suppl.

[CR101] Wolkoff P (2008). “Healthy” eye in office-like environments. Environ Int.

[CR102] Wolska A, Switula M (1999). Luminance of the surround and visual fatigue of VDT operators. Int J Occup Saf Ergon.

[CR103] Woods V (2005). Musculoskeletal disorders and visual strain in intensive data processing workers. Occup Med.

[CR104] Zetterberg C, Forsman M, Richter HO (2013). Effects of visually demanding near work on trapezius muscle activity. J Electromyogr Kinesiol.

[CR105] Zetterlund C, Lundqvist L-O, Richter HO (2009). The relationship between low vision and musculoskeletal complaints. A case control study between age-related macular degeneration patients and age-matched controls with normal vision. J Optometry.

[CR106] Zhang Q, Lindberg L-G, Kadefors R, Styf J (2001). A non-invasive measure of changes in blood flow in the human anterior tibial muscle. Eur J Appl Physiol.

